# Modulation of Rat Hepatic CYP1A and 2C Activity by Honokiol and Magnolol: Differential Effects on Phenacetin and Diclofenac Pharmacokinetics In Vivo

**DOI:** 10.3390/molecules23061470

**Published:** 2018-06-17

**Authors:** Sang-Bum Kim, Kyu-Sang Kim, Heon-Min Ryu, Seong-Ho Hong, Bo-Kyoung Kim, Dae-Duk Kim, Jin Woo Park, In-Soo Yoon

**Affiliations:** 1 New Drug Development Center, Daegu‒Gyeongbuk Medical Innovation Foundation, Daegu 41061, Korea; ksb2014@dgmif.re.kr (S.-B.K.); kbky9872@gmail.com (B.-K.K.); 2 College of Pharmacy and Research Institute of Pharmaceutical Sciences, Seoul National University, Seoul 08826, Korea; kyuritas00@naver.com (K.-S.K.); newrhm@snu.ac.kr (H.-M.R.); ddkim@snu.ac.kr (D.-D.K.); 3 Biomedicine Lab, CKD Research Institute, Gyeonggi 16995, Korea; duck1240@naver.com; 4 Department of Pharmacy, College of Pharmacy and Natural Medicine Research Institute, Mokpo National University, Jeonnam 58554, Korea; 5 Department of Manufacturing Pharmacy, College of Pharmacy, Pusan National University, Busan 46241, Korea

**Keywords:** honokiol, magnolol, *Magnolia officinalis*, CYP1A, CYP2C, rat, pharmacokinetics

## Abstract

Honokiol (2-(4-hydroxy-3-prop-2-enyl-phenyl)-4-prop-2-enyl-phenol) and magnolol (4-Allyl-2-(5-allyl-2-hydroxy-phenyl)phenol) are the major active polyphenol constituents of *Magnolia officinalis* (Magnoliaceae) bark, which has been widely used in traditional Chinese medicine (Houpu Tang) for the treatment of various diseases, including anxiety, stress, gastrointestinal disorders, infection, and asthma. The aim of this study was to investigate the direct effects of honokiol and magnolol on hepatic CYP1A and 2C-mediated metabolism in vitro using rat liver microsomes and in vivo using the Sprague-Dawley rat model. Honokiol and magnolol inhibited in vitro CYP1A activity (probe substrate: phenacetin) more potently than CYP2C activity (probe substrate: diclofenac): The mean IC_50_ values of honokiol for the metabolism of phenacetin and diclofenac were 8.59 μM and 44.7 μM, while those of magnolol were 19.0 μM and 47.3 μM, respectively. Notably, the systemic exposure (AUC and C_max_) of phenacetin, but not of diclofenac, was markedly enhanced by the concurrent administration of intravenous honokiol or magnolol. The differential effects of the two phytochemicals on phenacetin and diclofenac in vivo pharmacokinetics could at least be partly attributed to their lower IC_50_ values for the inhibition of phenacetin metabolism than for diclofenac metabolism. In addition, the systemic exposure, CL, and V_ss_ of honokiol and magnolol tended to be similar between the rat groups receiving phenacetin and diclofenac. These findings improve our understanding of CYP-mediated drug interactions with *M. officinalis* and its active constituents.

## 1. Introduction

The past two decades have seen a significant increase in the utilization of herbal extract formulations to complement prescription drugs administered for the prevention and treatment of disease [1]. Two independent national surveys have previously shown that the 12-month prevalence of herbal medicine use was 26.3% in England [2] and 18.9% in the United States [3]. Patients mainly tend to consume herbal medicine owing to dissatisfaction with the efficacy of prescription drugs, and/or the misconception that herbs are ‘natural’ and thus safer, than prescription drugs [4]. However, herbal extract products contain various active phytochemicals, some of which are cleared by the cytochrome P450 (CYP) isozymes responsible for the metabolism of most prescription drugs [5]. Thus, CYP-based herb-drug interactions are inevitable, yet the CYP-modulating potential and the associated in vivo pharmacokinetic consequences of many herbal extracts and their active constituents remain unexplored, necessitating further investigation [6].

**Figure 1:**
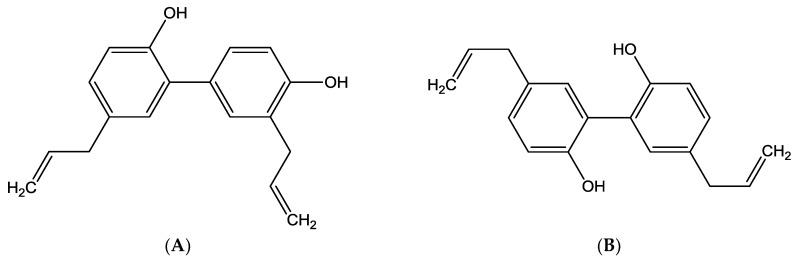
Chemical structures of honokiol (**A**) and magnolol (**B**).

*Magnolia officinalis* (Magnoliaceae) is mainly found in East Asia, and its bark has been widely used in traditional Chinese medicine (Houpu Tang) for the treatment of various diseases, including anxiety, stress, gastrointestinal disorders, infection, and asthma [7]. *M. officinalis* bark extract is commercially available as a dietary supplement in powder or capsule form [8]. Moreover, the supercritical carbon dioxide extract of *M. officinalis* bark is added to chewing gums to improve oral health by reducing salivary *Streptococcus mutans* levels [9]. Honokiol (2-(4-hydroxy-3-prop-2-enyl-phenyl)-4-prop-2-enyl-phenol) and magnolol (4-Allyl-2-(5-allyl-2-hydroxy-phenyl)phenol) (Figure 1) are the major active polyphenol constituents present in *M. officinalis* bark [10]. Both constituents have been shown to exert various common pharmacological effects, including anti-inflammatory, antioxidative, antidepressant-like, and neuroprotective activities [11,12,13,14,15,16]. It has been reported that humans could be regularly exposed to the two phytochemicals in their daily lives, through either the neutraceuticals or the chewing gum sources mentioned above [17].

Honokiol strongly inhibits CYP1A2 activity with half maximal inhibitory concentration (IC_50_) values of 2.1–4.7 μM, while it moderately or strongly inhibits CYP2B6, 2C8, 2C9, and 2C19 activity with IC_50_ values of 3.9–40.8 μM in human liver microsomes in vitro [18,19]. Magnolol also exerts potent inhibitory effects on CYP1A2, 2B6, and 2C9 activity with C_50_ values of 5.4–44.9 μM in human liver microsomes as well as CYP1A, 2C, and 3A activity with IC_50_ values of 1.62–35.0 μM in rat liver microsomes (RLM) in vitro [17,19]. Honokiol and magnolol could therefore potentially alter the in vivo pharmacokinetics of drugs that are the substrates of CYP1A and 2C isoforms. However, limited information is currently available regarding this issue, thereby warranting further investigation to improve our understanding of drug interactions with honokiol, magnolol, and *M. officinalis*.

In the present study, the direct effect of honokiol and magnolol on hepatic CYP1A and 2C-mediated metabolism was investigated in vitro using Sprague-Dawley (SD) RLM and in vivo using the SD rat model. The inhibitory potential of honokiol and magnolol on CYP activity in RLM was assessed to establish its IC_50_. The in vivo pharmacokinetics of phenacetin and diclofenac, respective probe substrates for rat CYP1A and 2C, with concurrent administration of a single intravenous dose of honokiol or magnolol, was evaluated in the SD rat model. Furthermore, the in vivo pharmacokinetics of intravenous honokiol or magnolol was also studied.

## 2. Results

### 2.1. Inhibitory Effect of Honokiol and Magnolol on Hepatic Metabolism of Phenacetin and Diclofenac

**Figure 2:**
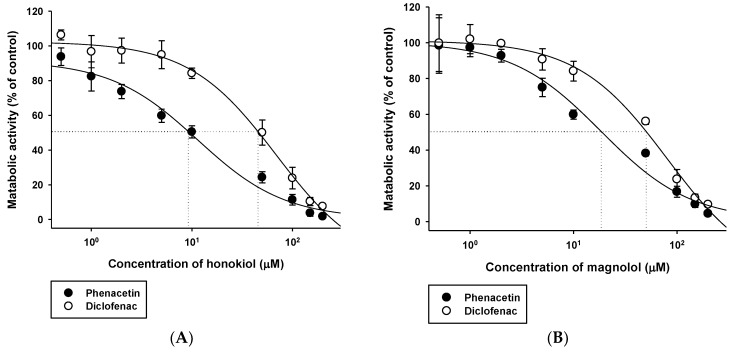
Effects of honokiol (**A**) and magnolol (**B**) on metabolic reactions of phenacetin (●) and diclofenac (○) in rat liver microsomes. The bullet symbols and their error bars represent the means and standard deviations, respectively (*n* = 4).

The effects of honokiol and magnolol on the disappearance rate of phenacetin and diclofenac were evaluated in RLM (Figure 2). The disappearance rate of phenacetin tended to be lower in the presence of honokiol and magnolol of ≥2 μM than in their absence. Similarly, the disappearance rate of diclofenac tended to be lower in the presence of ≥10 μM honokiol and ≥5 μM magnolol than in their absence. The dose-response curve was well described by the sigmoidal logistic equation (Equation (1); *R*^2^ = 0.964–0.995). The mean IC_50_ values of honokiol for the metabolism of phenacetin and diclofenac were 8.59 μM (2.2 μg/mL) and 44.7 μM (11.9 μg/mL), respectively (Hill coefficient = 0.98 and 1.49, respectively). The mean IC_50_ values of magnolol for the metabolism of phenacetin and diclofenac were 19.0 μM (5.1 μg/mL) and 47.3 μM (12.6 μg/mL), respectively (Hill coefficient = 0.97 and 1.41, respectively).

### 2.2. In Vivo Pharmacokinetics of Phenacetin and Diclofenac with or without Honokiol and Magnolol in Rats

**Figure 3:**
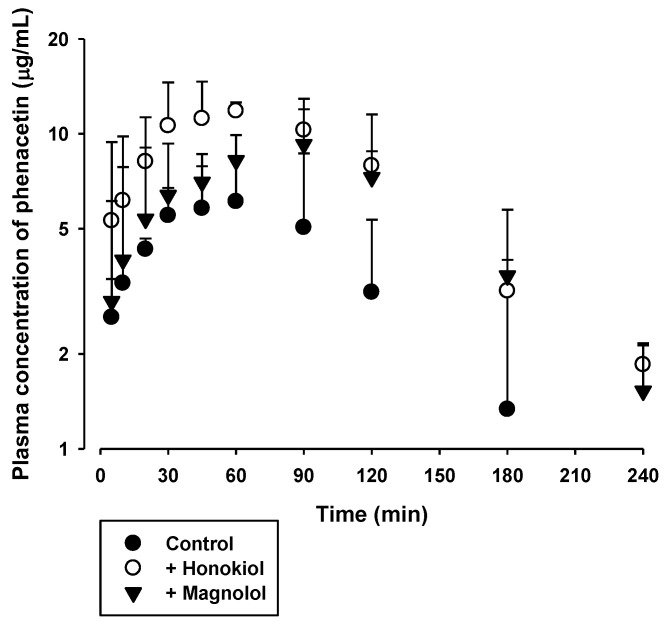
Plasma concentration versus time profiles of phenacetin in rats following its oral administration at a dose of 20 mg/kg without (●) or with 5 mg/kg intravenous honokiol (○) or magnolol (▼). The bullet symbols and their error bars represent the means and standard deviations, respectively (*n* = 4–5).

**Figure 4:**
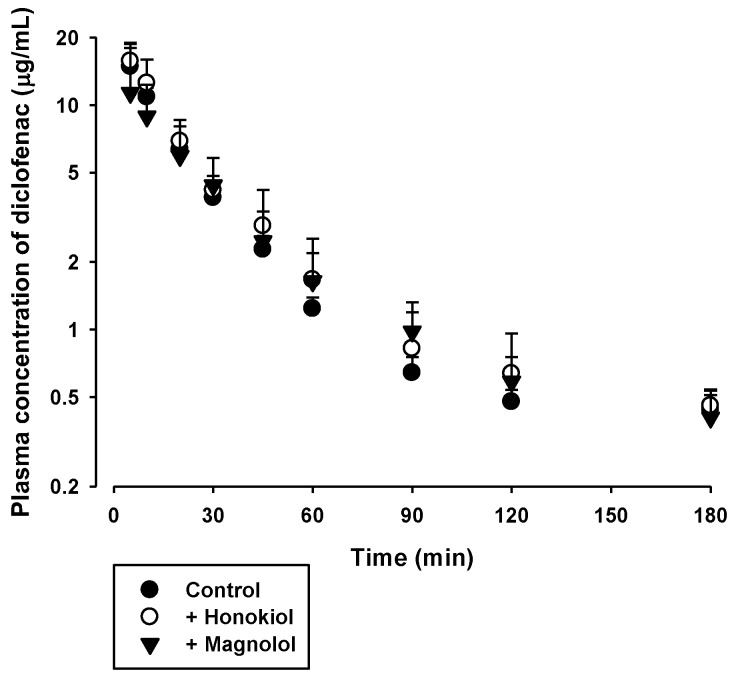
Plasma concentration versus time profiles of diclofenac in rats following its oral administration at a dose of 6 mg/kg without (●) or with 5 mg/kg intravenous honokiol (○) or magnolol (▼). The bullet symbols and their error bars represent the means and standard deviations, respectively (*n* = 4–5).

The effects of honokiol and magnolol on the in vivo pharmacokinetics of phenacetin and diclofenac were assessed. The plasma concentration versus time profiles of phenacetin and diclofenac, following oral administration with or without 5 mg/kg intravenous honokiol or magnolol in rats, are shown in Figure 3 and Figure 4, respectively. The relevant pharmacokinetic parameters are listed in Table 1 and Table 2, respectively. Compared to control rats, the AUC and C_max_ of orally administered phenacetin were markedly higher in rats with concurrent intravenous administration of honokiol (by 104%) or magnolol (by 78%). In contrast, the AUC and C_max_ of orally administered diclofenac tended to be similar among the three rat groups tested.

### 2.3. In Vivo Pharmacokinetics of Honokiol and Magnolol with Phenacetin and Diclofenac in Rats

**Figure 5:**
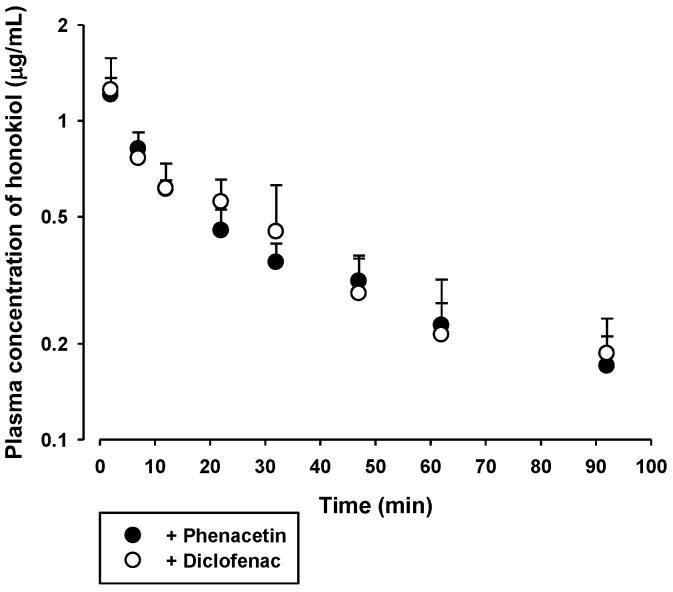
Plasma concentration versus time profiles of honokiol in rats following its intravenous injection at a dose of 5 mg/kg with concurrent administration of 20 mg/kg oral phenacetin (●) or 6 mg/kg diclofenac (○). The bullet symbols and their error bars represent the means and standard deviations, respectively (*n* = 4).

**Figure 6:**
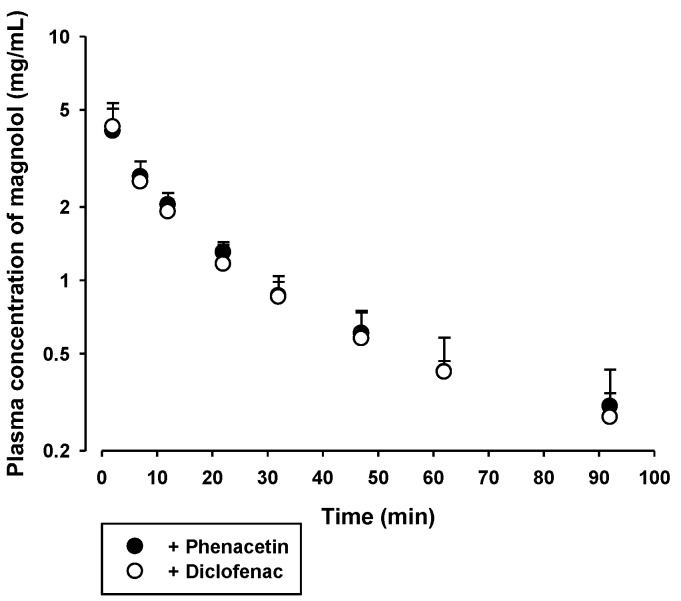
Plasma concentration versus time profiles of magnolol in rats following its intravenous injection at a dose of 5 mg/kg with concurrent administration of 20 mg/kg oral phenacetin (●) or 6 mg/kg diclofenac (○). The bullet symbols and their error bars represent the means and standard deviations, respectively (*n* = 4).

The in vivo pharmacokinetics of honokiol and magnolol with concurrent administration of phenacetin or diclofenac was assessed. The plasma concentration versus time profiles of honokiol and magnolol, following intravenous injection with concurrent oral administration of 20 mg/kg phenacetin or 6 mg/kg diclofenac in rats, are shown in Figure 5 and Figure 6, respectively. The relevant pharmacokinetic parameters are listed in Table 3 and Table 4, respectively. After intravenous dosing, the plasma levels of honokiol and magnolol showed multi-exponential decay with a terminal half-life of 20.3–28.5 min for honokiol and 30.8–50.4 min for magnolol. The AUC, CL, and V_ss_ of honokiol and magnolol tended to be similar between the two rat groups treated with phenacetin and diclofenac. The highest plasma levels of honokiol and magnolol observed at 2 min were 0.9–1.6 μg/mL and 3.1–5.5 μg/mL, respectively.

## 3. Discussion

The present study provides novel data on the pharmacokinetic interactions of honokiol and magnolol with the CYP1A and 2C substrate drugs, phenacetin and diclofenac, in the SD rat model. Currently, Magnolia bark extract (MBE) is commercially available as a dietary supplement in the form of a powder or capsule. For example, one of the MBE products (Swanson Superior Herbs Magnolia Extract (SWH225); Swanson Health Products, Fargo, ND, USA) is sold as a capsule formulation containing MBE standardized to 90% honokiol. Its daily dose is 200 mg, which corresponds to 3 mg/kg as honokiol in 60 kg human. For magnolol, it has been reported that daily exposure to magnolol derived from mints and gums (containing MBE) to remove oral maloder can reach 1.64 mg/kg in humans [20,21]. In general, maximum recommended starting dose (MRSD) in initial clinical trials can be estimated by converting animal dose to human equivalent dose (HED) using a scaling factor, followed by application of a safety factor (FDA guidance: Estimating the Maximum Safe Starting Dose in Initial Clinical Trials for Therapeutics in Adult Healthy Volunteers) [22]. Assuming the scaling based on body weight (i.e., HED (mg/kg) set equal to animal dose (mg/kg)) and the safety factor of 10 (default value), the daily oral doses of honokiol and magnolol in human can be converted to rat oral doses of 30 and 16.4 mg/kg, respectively. The oral bioavailability (F) values of honokiol and magnolol are estimated to be 23.2 and 32.3%, respectively, based on the results reported in previous rat intravenous and oral pharmacokinetic studies (see Table S1 in the Supplementary Information). Taking the F values into account, it is plausible that the above-mentioned rat oral doses (30 and 16.4 mg/kg) could provide a systemic exposure (AUC) equivalent to rat intravenous honokiol and magnolol doses of 6.9 and 5.3 mg/kg, respectively, which are comparable to the intravenous doses used in our present study (5 mg/kg). Additionally, the oral doses of phenacetin (20 mg/kg) and diclofenac (6 mg/kg), model drugs as probe substrates of CYP1A and CYP2C, respectively, in the present study were set below those used in previous pharmacokinetic drug interaction studies in rats [23,24]. Therefore, the drug/phytochemical doses and their interactions tested in the present study could have some implications and relevance to clinical settings, which warrant further systematic clinical study.

The inhibitory effect of honokiol and magnolol on the hepatic metabolism of the two respective drugs was assessed by measuring the drug disappearance rate in the presence of either honokiol or magnolol at various concentrations. As shown in Figure 2 and Figure 3, the IC_50_ values of honokiol and magnolol for phenacetin metabolism were markedly lower than those for diclofenac metabolism (honokiol: 8.59 μM versus 44.7 μM; magnolol: 19.0 μM versus 47.3 μM). These results suggest the inhibitory potential of honokiol and magnolol on CYP1A activity is more potent than on CYP2C activity in RLM. A previous study using human liver microsomes reported honokiol and magnolol to have more potent inhibitory effect on CYP1A2 (phenacetin as probe substrate; IC_50_ = 3.5 μM and 5.4 μM, respectively) than on CYP2C8 (amodiaquine as probe substrate; IC_50_ = 40.8 μM and >50 μM, respectively), CYP2C9 (tolbutamide as probe substrate; IC_50_ = 9.6 μM and 10.2 μM, respectively), and CYP2C19 (omeprazole as probe substrate; IC_50_ = 32.9 μM and >50 μM, respectively) [19], which is consistent with the present RLM data. Notably, the Hill coefficients for the inhibitory effect of honokiol and magnolol on the metabolism of phenacetin were close to 1 (0.98 for honokiol and 0.97 for magnolol) in the present study, which is expected for competitive inhibition [25].

To investigate the inhibitory effect of honokiol and magnolol on the hepatic metabolism of phenacetin and diclofenac, the pharmacokinetics of orally administered phenacetin and diclofenac with or without single concurrent administration of intravenous honokiol and magnolol was evaluated in the rat model. Phenacetin is eliminated primarily by the CYP1A2-mediated hepatic metabolism in rats and humans [26,27]. Its absolute oral bioavailability is approximately 45% in rats, owing to the hepatic first-pass metabolism [28,29]. Diclofenac is extensively metabolized by phase I and II reactions in the liver [24]. The hepatic phase I metabolism of diclofenac is mediated by human CYP2C9 and rat CYP2C11 [30]. The oral doses of phenacetin (20 mg/kg) and diclofenac (6 mg/kg) were selected based on previous rat pharmacokinetic studies [24,27,31]. Notably, the systemic exposure (AUC and C_max_) of phenacetin was markedly enhanced by the concurrent administration of either intravenous honokiol or magnolol (Figure 3 and Table 1), while that of diclofenac was not altered by the same treatment (Figure 4 and Table 2). As shown in Figure 2, the IC_50_ values indicate that honokiol and magnolol exert much higher in vitro inhibitory effects on phenacetin metabolism than on diclofenac metabolism. The plasma levels of honokiol and magnolol with concurrent intravenous phenacetin administration tended to be similar to those with diclofenac administration (Figure 5 and Figure 6). However, the cellular level of honokiol and magnolol in the rat liver is difficult to estimate from plasma concentration data, since the distribution coefficient of honokiol and magnolol in the rat liver is currently unknown. Thus, assuming the plasma concentrations of honokiol and magnolol directly relate to their concentrations in the rat liver (hepatocytes), it is plausible that the differential effects of the two phytochemicals on in vivo phenacetin and diclofenac pharmacokinetics could be attributed to their lower IC_50_ values for phenacetin metabolism than those for diclofenac metabolism.

The plasma levels of honokiol and magnolol at 2 min after intravenous administration were comparable to their IC_50_ values (2.0–5.1 μg/mL) for phenacetin metabolism, and those during the initial 12–22 min exceeded their IC_25_ values (approximately 0.5–1.3 μg/mL). Thus, assuming the distribution coefficient of honokiol and magnolol in the rat liver (hepatocyte) is 1 or above, it is possible that intravenous honokiol and magnolol doses could inhibit in vivo hepatic CYP1A-mediated phenacetin metabolism in rats. This is consistent with the observed elevated systemic exposure (AUC and C_max_) of orally administered phenacetin by concurrent administration of honokiol and magnolol (Figure 3 and Table 1). By contrast, the plasma levels of intravenous honokiol and magnolol were far below their IC_50_ (9.2–13.1 μg/mL) and IC_25_ values (approximately 2.7–13.3 μg/mL) for the inhibition of diclofenac metabolism. This result coincides well with the unaltered systemic exposure (AUC and C_max_) of orally administered diclofenac by concurrent honokiol and magnolol administration (Figure 4 and Table 2). In addition, our present pharmacokinetic data for honokiol and magnolol can be compared with previously reported rat pharmacokinetic parameters of honokiol and magnolol administered alone (Table S1). Comparison among Table 3, Table 4, and Table S1 indicates that most pharmacokinetic parameters of honokiol and magnolol following intravenous administration of either honokiol or magnolol alone do not seem to differ much from those with concurrent administration of phenacetin and diclofenac. However, the V_ss_ values of honokiol administered alone tended to be markedly lower than those with concurrent administration of phenacetin and diclofenac. This may be attributed to the alteration of drug-protein binding in blood or tissues, which requires further investigation to clarify its exact mechanism.

Since oral MBE formulation only is currently available in the market, oral dosing of honokiol and magnolol seems more relevant to the current clinical setting. However, as discussed above, the intravenous dosing used in this study could also have some clinical implications, based on the MRSD-HED-animal dose conversion and oral bioavailability concepts. Moreover, several preclinical studies demonstrated various pharmacological efficacies of intravenous honokiol and magnolol treatment [32,33,34], which can lead us to expect the clinical development of intravenous formulation of honokiol, magnolol, or MBE in the near future. In the current state, our present results showed the feasibility of honokiol and magnolol to modulate CYP activity in vivo, potentially providing useful information for the development of herbal medicine containing honokiol, magnolol, and/or MBE.

## 4. Materials and Methods

### 4.1. Materials

Diclofenac, honokiol, magnolol, phenacetin, and the reduced form of β-nicotinamide adenine dinucleotide phosphate (NADPH; as a tetrasodium salt) were purchased from Sigma-Aldrich Co. (St. Louis, MO, USA). The purity of all purchased compounds was higher than 98.0%. RLM was purchased from BD-Genetech (Woburn, MA, USA). Other chemicals were of reagent grade or high-performance liquid chromatography (HPLC) grade.

### 4.2. In Vitro CYP Inhibition study in RLM

An in vitro CYP inhibition study using RLM was conducted in accordance with the manufacturer’s protocol using BD-Gentest™ pooled male RLM (SD rats). The microsomal incubation mixture, comprising RLM (0.5-mg/mL microsomal protein), 1 mM NADPH, 10 mM MgCl_2_, 50 mM potassium phosphate buffer, substrate (10 μM phenacetin or 5 μM diclofenac), and inhibitor (0, 0.5, 1, 2, 5, 10, 50, 100, 150, and 200 μM honokiol or magnolol), was prepared in a total volume of 0.5 mL. The disappearance of substrate in the absence or presence of inhibitor was determined to assess the effect of inhibitor on the CYP-mediated metabolism of substrate in RLM. Metabolic reactions were initiated by the addition of each substrate, and incubation was conducted at 37 °C in a shaking water bath. After incubation for 0 and 20 min, a 100 μL aliquot of the microsomal incubation mixture was sampled and transferred into a clean 1.5 mL microcentrifuge tube containing 100 μL cold acetonitrile to terminate the metabolic reaction. After vortex mixing and centrifugation at 16,000× *g* for 10 min, a 100 μL aliquot of the supernatant was stored at −80 °C until HPLC analysis.

### 4.3. Animals

Protocols for the animal studies were handled in accordance with the guidelines for the Institutional Animal Care and Use Committee of Seoul National University (SNU-160311-3-1). Male SD rats (7–9 weeks old) were purchased from Samtako Bio Korea (Osan, South Korea). They were acclimatized in the university animal facility at 20–23 °C with 12 h light (07:00–19:00) and dark (19:00–07:00) cycles, and a relative humidity of 50% ± 5%. After acclimatization, the rats of >300 g body weight were used for the pharmacokinetic study. The rats were housed in metabolic cages (Tecniplast USA, Inc., Chester, PA, USA) under filtered, pathogen free air, with food (Agribrands Purina Korea, Inc., Seongnam, Korea) and water available *ad libitum*. The rats were fasted overnight before the oral pharmacokinetic study.

### 4.4. In Vivo Pharmacokinetic Study in Rats

Following anesthetization by intramuscular injection of zoletil at 20 mg/kg, the rats’ femoral vein and artery were cannulated with a polyethylene tube (Clay Adams) 4 h before drug administration. Rats were administered a single oral administration of phenacetin (20 mg/kg; dissolved in 100% PEG400) or diclofenac (6 mg/kg; dissolved in saline) with or without a single concurrent intravenous dose of honokiol or magnolol at 5 mg/kg (dissolved in a vehicle consisting of PEG400, ethanol, and saline at the ratio of 2:1:4, *v/v/v*). An aliquot of approximately 200 μL of blood was collected via the femoral artery at 0, 5, 10, 20, 30, 45, 60, 90, 120, 180, and 240 min after the oral dose of phenacetin or diclofenac, and at 0, 2, 7, 12, 22, 32, 47, 62, and 92 min after the intravenous dose of honokiol or magnolol. Following centrifugation of the blood sample at 2000× *g* at 4 °C for 10 min, a 75 μL aliquot of plasma was stored at −80 °C until HPLC analysis.

### 4.5. HPLC Analysis

The concentrations of drugs (phenacetin and diclofenac) and phytochemicals (honokiol and magnolol) in the microsomal and/or plasma samples were determined as previously reported with slight modifications [35,36,37,38]. A 75 μL aliquot of the sample was first deproteinized with 200 μL acetonitrile containing internal standard (IS; tolbutamide for phenacetin; diflunisal for diclofenac; magnolol for honokiol, honokiol for magnolol). Following vortex mixing and centrifugation at 16,000× *g* at 4 °C for 10 min, the supernatant was transferred to a clean 1.5 mL microcentrifuge tube, and dried under nitrogen gas at 25 °C. The residue was reconstituted with 100 μL mobile phase and a 50 μL aliquot was injected into a reversed-phase HPLC column (C18 Gemini NX; 250 mm length × 4.6 mm i.d.; particle size 5 μm; Phenomenex). The mobile phase was a mixture of 10 mM phosphate monobasic solution (pH 2.4, solvent A) and acetonitrile (solvent B). For phenacetin, the following gradient system was used: 70% (*v*/*v*) to 30% (*v*/*v*) solvent A during 0–8 min; and 70% (*v*/*v*) solvent A during 8–11 min. For diclofenac, the following gradient system was used: 81% (*v*/*v*) to 18% (*v*/*v*) solvent A during 0–6 min; and 45% (*v*/*v*) solvent A during 6–8 min. For honokiol, the isocratic-containing solvent A of 70% (*v*/*v*) was used. For magnolol, the following gradient system was used: 45% (*v*/*v*) to 15% (*v*/*v*) solvent A during 0–6 min; and 45% (*v*/*v*) solvent A during 6–8 min. The flow rate of the mobile phase was 1.0 mL/min, and the column effluent was monitored by a UV/Vis detector at 245 nm for phenacetin, 254 nm for diclofenac, 290 nm for honokiol, and 280 nm for magnolol at 25 °C.

### 4.6. Data Analysis

Standard methods were used to calculate the following pharmacokinetic parameters using a non-compartmental analysis (WinNonlin version 3.1; Certara USA, Inc., Princeton, NJ, USA): The total area under the plasma concentration versus time curve from time zero to time infinity (AUC); the time-averaged total body clearance (CL, calculated as dose/AUC); the total area under the first moment of plasma concentration versus time curve (AUMC); the apparent volume of distribution at steady state (V_ss_, calculated as dose × AUMC/AUC^2^); and the terminal half-life (t_1/2_) [39]. The peak plasma concentration (C_max_) and time to reach C_max_ (T_max_) were read directly from the experimental data. The relative IC_50_ of honokiol and magnolol for the inhibition of phenacetin or diclofenac metabolism was determined by nonlinear regression using GraphPad Prism (version 5.01; GraphPad Software, San Diego, CA, USA), according to the following four-parameter logistic equation [5]:

$$Y=\mathrm{Min}+\;\frac{\mathrm{Max}-\mathrm{Min}}{1+{\left(\frac{X}{{\mathrm{IC}}_{50}}\right)}^{-p}}$$

where X and Y are the inhibitor concentration and response, respectively. Max and Min are the initial and final Y values, respectively, and the power P represents the Hill coefficient.

### 4.7. Statistical Analysis

Only descriptive statistics was used in this study (*n* = 4 or 5). All data were expressed as mean ± standard deviation, except for median ranges for T_max_, and rounded to one decimal place.

## 5. Conclusions

The present study demonstrates that honokiol and magnolol inhibit CYP1A activity more potently than CYP2C activity in vitro. Similarly, the in vivo pharmacokinetics of orally administered phenacetin was markedly altered by concurrent administration of the two phytochemicals, while the in vivo pharmacokinetics of orally administered diclofenac remained unaffected. To the best of our knowledge, this is the first report on the differential effects of honokiol and magnolol on the in vitro hepatic metabolism of phenacetin and diclofenac in RLM as well as their in vivo pharmacokinetic consequences in rats. These results provide an improved understanding of CYP-mediated drug interactions with *M. officinalis* and its active constituents.

## Figures and Tables

**Table 1:** Pharmacokinetic parameters of phenacetin in rats following its oral administration at a dose of 20 mg/kg with or without 5 mg/kg intravenous honokiol or magnolol (*n* = 4–5).

Parameter	Control	+ Honokiol	+ Magnolol
AUC (μg·min/mL)	820 ± 374	1670 ± 343	1460 ± 100
T_1/2_ (min)	48.7 ± 38.7	46.0 ± 15.3	64.9 ± 22.3
C_max_ (μg/mL)	6.67 ± 1.91	13.0 ± 1.7	10.1 ± 1.8
T_max_ (min)	60 (30–90)	60 (45–90)	90 (30–90)

**Table 2:** Pharmacokinetic parameters of diclofenac in rats following its oral administration at a dose of 6 mg/kg with or without 5 mg/kg intravenous honokiol or magnolol (*n* = 4–5).

Parameter	Control	+ Honokiol	+ Magnolol
AUC (μg·min/mL)	430 ± 18	496 ± 99	435 ± 81
T_1/2_ (min)	49.8 ± 16.7	69.1 ± 26.9	69.4 ± 19.1
C_max_ (μg/mL)	14.9 ± 3.1	15.7 ± 3.0	12.0 ± 6.9
T_max_ (min)	5	5	7.5 (5–10)

**Table 3:** Pharmacokinetic parameters of honokiol in rats following its intravenous injection at a dose of 5 mg/kg with 20 mg/kg oral phenacetin or 6 mg/kg diclofenac (*n* = 4).

Parameter	+ Phenacetin	+ Diclofenac
AUC (μg·min/mL)	48.2 ± 9.5	50.1 ± 12.7
T_1/2_ (min)	51.5 ± 3.5	48.0 ± 3.4
CL (mL/min/kg)	106 ± 21	104 ± 23
V_ss_ (mL/kg)	7080 ± 1180	6570 ± 928

**Table 4:** Pharmacokinetic parameters of magnolol in rats following its intravenous injection at a dose of 5 mg/kg with 20 mg/kg oral phenacetin or 6 mg/kg diclofenac (*n* = 4).

Parameter	+ Phenacetin	+ Diclofenac
AUC (μg·min/mL)	110 ± 6	102 ± 31
T_1/2_ (min)	41.3 ± 10.9	32.7 ± 9.8
CL (mL/min/kg)	45.5 ± 2.7	52.2 ± 14.6
V_ss_ (mL/kg)	2040 ± 226	1880 ± 341

## Supplementary Materials

The following are available online at http://www.mdpi.com/1420-3049/23/6/1470/s1, Table S1: Pharmacokinetic parameters of honokiol and magnolol reported in previous literatures on rat intravenous and oral pharmacokinetic studies.
