# Supplementary material for: Modulation of Rat Hepatic CYP1A and 2C Activity by Honokiol and Magnolol: Differential Effects on Phenacetin and Diclofenac Pharmacokinetics In Vivo
Source: Molecules. 2018 Jun 17;23(6):1470. doi: 10.3390/molecules23061470 (PMC6100004; doi:10.3390/molecules23061470)
Supplement: Supplementary file 1 [file molecules-23-01470-s001.pdf]

Molecules

Supplementary Information

Modulation of Rat Hepatic CYP1A and 2C Activity by Honokiol and Magnolol: Differential Effects on Phenacetin and Diclofenac Pharmacokinetics In Vivo

Sang-Bum Kim, Kyu-Sang Kim, Heon-Min Ryu, Seong-Ho Hong, Bo-Kyoung Kim, Dae-Duk Kim, Jin Woo Park and In-Soo Yoon

Table S1. Pharmacokinetic parameters of honokiol and magnolol reported in previous literatures on rat intravenous and oral pharmacokinetic studies.

Parameter	Honokiol		Magnolol	
	IV	PO	IV	PO
Dose (mg/kg)	5	41.4	5	56.3
AUC (μg·min/mL)	58.87	113.28	173.56	631.44
CL (mL/min/kg)	86.16		27.05	
V_{ss} (mL/kg)	2542		1619	
C_{max} (μg/mL)		1.026		6.769
F (%)		23.2		32.3
Reference	[1]	[2]	[3]	[2]

References for Supplementary Information

1. Tsai, T. H.; Chou, C. J.; Cheng, F. C.; Chen, C. F. Pharmacokinetics of honokiol after intravenous administration in rats assessed using high-performance liquid chromatography. *J Chromatogr. B* **1994**, 655, 41-45.
2. Hu, H.; Wang, Z.; Hua, W.; You, Y.; Zou, L. Effect of chemical profiling change of processed *Magnolia officinalis* on the pharmacokinetic profiling of honokiol and magnolol in rats. *J. Chromatogr. Sci.* **2016**, 54, 1201-1212.
3. Tsai, T. H.; Chou, C. J.; Chen, C. F. Pharmacokinetics and brain distribution of magnolol in the rat after intravenous bolus injection. *J. Pharm. Pharmacol.* **1996**, 48, 57-59.
